# Investigation of 91 proteins implicated in neurobiological processes identifies multiple candidate plasma biomarkers of stroke outcome

**DOI:** 10.1038/s41598-022-23288-5

**Published:** 2022-11-22

**Authors:** Cecilia Lagging, Sofia Klasson, Annie Pedersen, Staffan Nilsson, Katarina Jood, Tara M. Stanne, Christina Jern

**Affiliations:** 1 Department of Laboratory Medicine, Institute of Biomedicine, The Sahlgrenska Academy, University of Gothenburg, Box 440, 405 30 Gothenburg, Sweden; 2 Department of Clinical Genetics and Genomics, Sahlgrenska University Hospital, Region Västra Götaland, Gothenburg, Sweden; 3 Division of Applied Mathematics and Statistics, Department of Mathematical Sciences, Chalmers University of Technology, Gothenburg, Sweden; 4 Department of Clinical Neuroscience, Institute of Neuroscience and Physiology, The Sahlgrenska Academy, University of Gothenburg, Gothenburg, Sweden; 5 Department of Neurology, Sahlgrenska University Hospital, Region Västra Götaland, Gothenburg, Sweden

**Keywords:** Stroke, Outcomes research, Biomarkers

## Abstract

The inter-individual variation in stroke outcomes is large and protein studies could point to potential underlying biological mechanisms. We measured plasma levels of 91 neurobiological proteins in 209 cases included in the Sahlgrenska Academy Study on Ischemic Stroke using a Proximity Extension Assay, and blood was sampled in the acute phase and at 3-month and 7-year follow-ups. Levels were also determined once in 209 controls. Acute stroke severity and neurological outcome were evaluated by the National Institutes of Health Stroke Scale. In linear regression models corrected for age, sex, and sampling day, acute phase levels of 37 proteins were associated with acute stroke severity, and 47 with 3-month and/or 7-year outcome at false discovery rate < 0.05. Three-month levels of 8 proteins were associated with 7-year outcome, of which the associations for BCAN and Nr-CAM were independent also of acute stroke severity. Most proteins followed a trajectory with lower levels in the acute phase compared to the 3-month follow-up and the control sampling point. Conclusively, we identified multiple candidate plasma biomarkers of stroke severity and neurological outcome meriting further investigation. This study adds novel information, as most of the reported proteins have not been previously investigated in a stroke cohort.

## Introduction

There is a need for improved understanding of the biology underlying the large inter-individual variation in ischemic stroke outcomes. Indeed, while some individuals fully recover others are left with persistent severe disability, and part of this variation remains unexplained by clinical factors alone^1^. Exploratory searches for proteins related to stroke severity and/or outcomes could point towards candidate molecular mechanisms meriting further investigation. Proteins with neurobiological functions hold experimental promise as biomarkers of ischemic stroke severity and outcomes. However, few have yet been investigated in clinical studies which mainly have focused on circulating concentrations of inflammatory, cardiac, natriuretic and endocrine markers, e.g. C-reactive protein, interleukin 6, B-type natriuretic peptide (BNP), N-terminal pro-BNP (NT-proBNP), copeptin, and insulin-like growth factor 1 (IGF-1)^2–4^. Recently, our group and others have shown that higher circulating concentrations of neurofilament light chain (NfL, a specific marker of neuroaxonal damage) is associated with increasing clinical stroke severity^5^, infarct size^6,7^ and poor stroke outcome^5–7^. Moreover, we and others have found that levels of brain-derived neurotrophic factor (BDNF, a marker of neuronal plasticity), is lower in the acute stroke phase and that low concentrations are associated with poor short-term^8,9^ and long-term outcome^10^.

In light of the above findings, we wanted to explore a larger number of proteins with neurobiological functions for association with stroke severity as well as both short and long-term outcome after ischemic stroke. To broadly profile plasma levels, we used a commercially available multiplex, 91-protein panel (Olink® Neurology Panel) targeting proteins involved in neurobiological processes, including neurogenesis, axon guidance, regulation of neuronal death, immune response and angiogenesis. Our goal was to inform future clinical studies of candidate protein markers of stroke severity and outcomes, as well as mechanistic studies on stroke-induced neurological damage and repair. Herein, we report the results of our exploratory analysis of these proteins for association with stroke severity and outcome in 209 ischemic stroke survivors.

## Methods

### Study population and baseline characteristics

This study comprises participants in the longitudinal part of the Sahlgrenska Academy Study on Ischemic Stroke (SAHLSIS) and their matched controls. In the present sub-study, we restricted inclusion to cases with a complete series of blood sampling during follow-up (n = 209), i.e. with available samples from the acute phase, and follow-up visits at both 3 months and 7 years post-stroke. The design of SAHLSIS and the sub-study with 7-year follow-up has been described in detail^11,12^. In brief, patients presenting with first-ever or recurrent acute ischemic stroke at age 18–69 years were recruited consecutively at the stroke unit at the Sahlgrenska University Hospital, Gothenburg, Sweden during 1998–2003. For each case one control matched for age, sex and geographical region, was recruited by random selection from population-based cohorts or registers as described^11^. Controls were invited to one study visit and were included if they had no history or signs of cerebrovascular and/or cardiovascular disease. Both cases and controls were thoroughly characterized regarding cardiovascular risk factors and comorbidities as described^11^. Cardiovascular risk factors were defined as follows; hypertension: pharmacological treatment for hypertension and/or systolic blood pressure ≥ 160 mmHg, and/or diastolic blood pressure ≥ 90 mmHg; diabetes mellitus: dietary or pharmacological treatment and/or fasting plasma glucose ≥ 7.0 mmol/L; hyperlipidemia: pharmacological treatment, total fasting serum cholesterol > 5.0 mmol/L, and/or LDL > 3.0 mmol/L; smoking: current smoking as opposed to never or former (cessation at least one year from inclusion). Index strokes were categorized according to the Oxfordshire Community Stroke Project (OCSP) classification^13^, into: total anterior circulation infarct (TACI), partial anterior circulation infarct (PACI), posterior circulation infarct (POCI), and lacunar infarct (LACI). Acute stroke severity in cases was defined as the worst score according to the Scandinavian Stroke Scale (SSS) within the first 7 days after onset. The SSS is similar to the National Institutes of Health Stroke Scale (NIHSS), and was used more commonly in Sweden at the time of participant inclusion. To facilitate comparisons, all SSS scores in the present study have been converted to NIHSS scores using an algorithm^14^, and stroke severity will henceforth refer to these calculated NIHSS scores.

### Follow-up and outcomes

Surviving cases were invited to follow-up visits at 3 months and 7 years after index stroke. At both visits, the degree of neurological impairments was assessed by a stroke neurologist (KJ, CJ, Petra Redfors or Lukas Holmegaard); at the 3-month visit according to the SSS and at the 7-year visit according to the NIHSS. The 3-month SSS scores were converted to NIHSS scores as described above. Functional outcome was assessed according to the modified Rankin Scale (mRS). We chose the degree of neurological impairments according the NIHSS as the primary outcome measure because of the closer relation to the underlying biology of neurological damage and repair compared to measures of functional outcome that also reflect social factors and comorbidities^15^, but included the mRS as a secondary outcome for comparisons. At the 7-year follow-up, presence of cardiovascular risk factors was reassessed through questionnaires. Recurrent strokes during follow-up were identified through national registers and questionnaires, and events were verified by review of medical records as described^5^. Participants with a neurological comorbidity with plausible influence on protein measurements or outcome were identified through national registers and questionnaires as described^5^.

### Blood sampling and measurements of plasma protein levels

Blood samples were drawn during the hospital stay for ischemic stroke (median 4 days after index stroke, interquartile range (IQR) 3–6 days), at the 3-month visit (median 98 days, IQR 93–109 days), and at the 7-year visit (median 7.5 years, IQR − 5.9 to + 5.6 weeks). In controls, blood samples were drawn once at inclusion. On all occasions, venous blood was collected between 8.30 and 10.30 a.m. after an overnight fast. Blood was drawn in tubes containing 10% by volume ethylenediaminetetraacetic acid (EDTA). Plasma was isolated within 2 h by centrifugation at 2,000 × g at 4 °C for 20 min, aliquoted, and stored at − 80 °C pending analysis.

Levels of 91 proteins with documented or suggested involvement in neurology-related diseases and biological processes such as axon development, neurogenesis and synapse assembly were measured by the Proximity Extension Assay (PEA) technology in a commercially available multiplex panel (Olink® Neurology Panel, Uppsala, Sweden; www.olink.com). Of note the version of the panel used here did not contain NfL. All investigated proteins, including their Uniprot ID, encoding gene, and tissue with highest expression (GTEx), are listed in Supplemental Table S1. The PEA analysis has been described in detail elsewhere^16^. In brief, each protein is specifically targeted by one pair of antibodies labeled with complementary oligonucleotides. Upon specific antibody-antigen binding, the complementary oligonucleotides are allowed to hybridize by close proximity. By subsequent DNA polymerase extension, a protein specific target sequence is generated, which in turn is quantified by standard real-time polymerase chain reaction (PCR). PCR readouts are normalized by an extension control added to each sample and an inter-plate control on each plate. The final PEA output is given as Normalized Protein eXpression (NPX) values, an arbitrary unit given on a log2 scale where a higher value corresponds to a higher protein level. Of note, the NPX levels correspond to relative levels, i.e. absolute protein concentrations are not determined.

All analyses were performed by a board-certified laboratory technician at Olink, Uppsala, blinded to the clinical information. To minimize impact of inter-run variability, the three samples from each case were placed on the same plate together with the single sample from the matched control. Out of 836 samples analysed in total, 12 samples (from 5 cases, and 6 controls) did not pass quality control and were excluded from further analysis, corresponding to an assay success rate of > 98%. Seven proteins had at least one sample read-out below the limit of detection: Granulocyte colony-stimulating factor (G-CSF): 10 readouts; Leukocyte-associated immunoglobulin-like receptor 2 (LAIR-2): 10 readouts; Linker for activation of T-cells family member 1 (LAT): 4 readouts; Bone morphogenetic protein 4 (BMP-4): 3 readouts; Mitochondrial hydroxyacylglutathione hydrolase (HAGH): 3 readouts; Sialoadhesin (SIGLEC1): 1 readout; Glial cell line-derived neurotrophic factor (GDNF): 1 readout. For these 32 readouts in total, protein levels were set to the lowest limit of detection.

We calculated intra- and inter-run coefficients of variance (CV) for each protein by repeated measures of two pooled samples, that each was aliquoted in two wells on all analyzed plates (n = 10). Mean intra- and inter-run CVs were 6.5% and 11.5%, respectively. Intra-run CV exceeded 10% for one protein: G-CSF (13.7%). Inter-run CV exceeded 15% for 5 proteins: N-acylethanolamine-hydrolyzing acid amidase (NAAA), 27.3%; Neuropilin-2 (NRP2), 17.3%; C-type lectin domain family 10-member A (CLEC10A), 16.3%; G-CSF, 16.2% and Cytotoxic and regulatory T-cell molecule (CRTAM), 15.6%. For NAAA, the high inter-run CV was due to high variability in one of the 4 pooled aliquots, indicating technical errors for this protein in this aliquot only. All of these proteins were included in further analyses, but their results should be viewed with some caution.

### Statistical analyses

Differences were compared with χ^2^ test for categorical variables and with paired samples *t*-test for protein levels (NPX). Correlations between protein levels and clinical variables were assessed by Spearman’s rank correlation, and the variance in protein levels explained by age, sex and acute stroke severity was investigated by linear regression. Proteins were clustered hierarchically based on degree of covariation according to Euclidean distance. In multivariable analyses, NIHSS was treated as a continuous scale and linear regression was used to analyze associations between Normalized Protein eXpression (NPX) levels and the primary outcome (NIHSS). For the secondary outcome analyses, logistic regression was used to investigate association between NPX levels and poor functional outcome (mRS > 2). As the NPX value is given on a log2 scale, the yielded β-value/odds ratio corresponds to the predicted change in outcome per each doubling of protein levels. In both linear and logistic regression analyses of outcomes, pre-specified adjustments for age, sex, and day of blood sampling were made in model 1, with additional adjustment for acute stroke severity in model 2. Linear regression analyses were rerun in a sensitivity analysis where we excluded cases with recurrent stroke between inclusion and the 7-year follow-up or a neurological comorbidity (n = 29). The correlation between NIHSS score and infarct size might depend on infarct location, i.e. anterior circulation non-lacunar strokes as opposed to posterior or lacunar infarcts, which in turn could influence protein associations to the NIHSS. Therefore, we additionally performed analyses stratified by the OCSP groups TACI or PACI (n = 67) compared to POCI or LACI (n = 139) in the acute-phase and also at the 3-month follow-up.

Participants with missing information (hyperlipidemia: 4 cases; diabetes: 1 control; protein measurements passing quality control: 2 cases [acute phase], 3 cases [3 months], 1 case [7-years], 6 controls; time until blood draw: 2 cases [acute phase], 1 case [3 months]; and 1 case for 7-year NIHSS score) were excluded in the statistical tests including the specific variable with missing value.

In the outcome models and the paired samples *t*-tests, control for multiple testing by number of investigated proteins (i.e. n = 91) was done by false discovery rate (FDR)^17^. In these analyses, associations with FDR < 0.05 were considered statistically significant, and associations with FDR ≥ 0.05 but a two-tailed *p* < 0.05 were considered suggestive. In remaining analyses, a two-tailed *p* < 0.05 was considered statistically significant. Statistical analyses were performed in IBM SPSS Statistics 26 and R version 4.0.5.

### Ethical approval

This study was approved by the Regional Ethics Review Board in Gothenburg and performed in accordance with the 1964 Helsinki Declaration. Written informed consent was obtained from all participants prior to enrollment. For participants who were unable to communicate, consent was obtained from their next-of-kin.

## Results

Baseline characteristics for the study population are summarized in Table 1. The median age at inclusion was 55 years (range 18–69 years), 65% were males, and as expected, cardiovascular risk factors were more prevalent among ischemic stroke cases than controls. None of the cases received acute recanalization therapy (intravenous thrombolysis or thrombectomy). Most cases experienced a mild stroke, but acute stroke severity ranged up to a maximum of 25 (NIHSS). The distribution of OCSP subtypes was relatively even, with the exception of total anterior circulation infarcts in only 12 cases (6%).

**Table 1:** Baseline characteristics.

	Ischemic stroke cases (n = 209)	Controls (n = 209)
Age years, median (IQR)	55 (49–61)	55 (49–61)
Male, n (%)	135 (64.6)	135 (64.6)
Hypertension, n (%)	111 (53.1) ***	68 (32.5)
Hyperlipidemia, n (%)	150 (73.1)^a^ **	125 (59.8)
Diabetes mellitus, n (%)	30 (14.4) *	14 (6.7)^b^
Current smoker, n (%)	73 (34.9) **	44 (21.1)
Previous history of stroke, n (%)	32 (15.3)	NA
Acute NIH stroke scale score, median (IQR)	2.5 (1.2–5.0)	NA
Index stroke OCSP subtype		NA
TACI, n (%)	12 (5.7)	
PACI, n (%)	55 (26.3)	
POCI, n (%)	71 (34.0)	
LACI, n (%)	68 (32.5)	
Unclassified, n (%)	3 (1.4)	

Difference in categorical variables between cases and the age and sex matched controls were tested by χ^2^ test. *IQR* Interquartile range. *NA* Not applicable. *NIH* National Institutes of Health. *OCSP* Oxfordshire Community Stroke Project. *TACI* Total anterior circulation infarct. *PACI* Partial anterior circulation infarct. *POCI* Posterior circulation infarct. *LACI* Lacunar infarct.

^a^Four cases with missing information. ^b^One control with missing information.

* *p* < 0.05, ** *p* < 0.01, *** *p* < 0.001.

At follow-up, NIHSS scores ranged up to a maximum of 16 at the 3-month follow-up, and 18 at the 7-year visit, with a majority of cases scoring NIHSS = 0 at both time points (60% at the 3-month and 65% at the 7-year follow-up). Median mRS score was 2 at both the 3-month (range mRS 0–4) and 7-year (range mRS 0–5) follow-ups, corresponding to 25 cases (12%) with poor functional outcome (mRS > 2) at 3 months and 30 (14%) at 7 years. During the 7-year follow-up 25 cases experienced a recurrent stroke and 6 cases were diagnosed with a neurological comorbidity with plausible influence on outcome or protein levels (including basal ganglia and autoimmune nervous system diseases), whereof 2 cases experienced both.

### Acute phase protein levels and associations with stroke severity and neurological outcome

**Figure 1:**
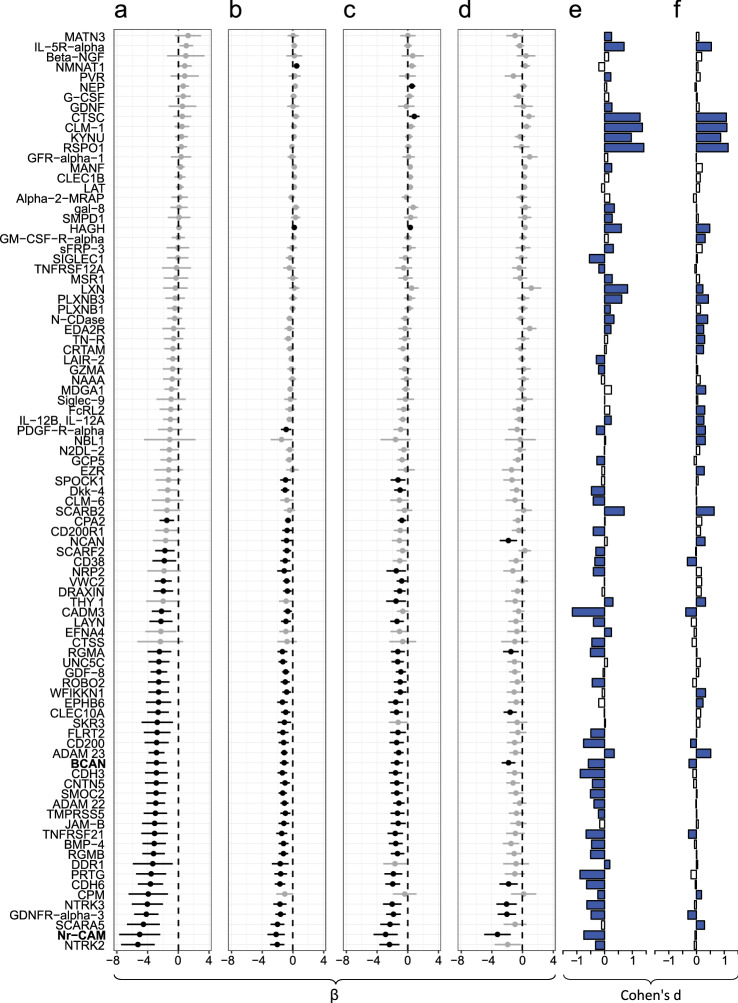
Associations between plasma protein levels, acute stroke severity and short- and long-term neurological outcome in 209 ischemic stroke cases, and comparisons of plasma protein levels between these cases and controls at the different time-points. Panels (**a**–**d**): Associations between plasma protein levels (Normalized Protein eXpression [NPX] levels) and degree of neurological impairments according to the NIH stroke scale (NIHSS), visualized with forest plots with β values and 95% confidence intervals derived from multivariable linear regression models adjusted for age, sex, and time until the blood draw (model 1). Associations with false discovery rate (FDR) < 0.05 are marked in black. As the NPX value is given on a log2 scale, the yielded β-value corresponds to the predicted change in NIHSS score per each doubling of protein levels*.* Associations between: (**a**) plasma protein levels and NIHSS score in the acute stroke phase, (**b**) plasma protein levels in the acute phase and NIHSS score at 3 months post-stroke, (**c**) plasma protein levels in the acute phase and NIHSS score at 7 years post-stroke, (**d**) plasma protein levels at 3 months and NIHSS score at 7 years post-stroke. For BCAN and Nr-CAM (marked in bold), the association between 3-month levels and 7-year outcome withstood additional adjustment for stroke severity. Panels (**e–f**): Differences in plasma protein levels between cases and controls. The bars correspond to Cohen’s d, i.e. the mean difference in case and control levels divided by the standard deviation for the controls, where a positive value indicates higher levels in cases than controls. Blue bars correspond to comparisons with a statistically significant difference (FDR < 0.05). (**e**) Comparison between acute phase samples (cases) and controls. (**f**) Comparison between 3-month samples (cases) and controls.

**Figure 2:**
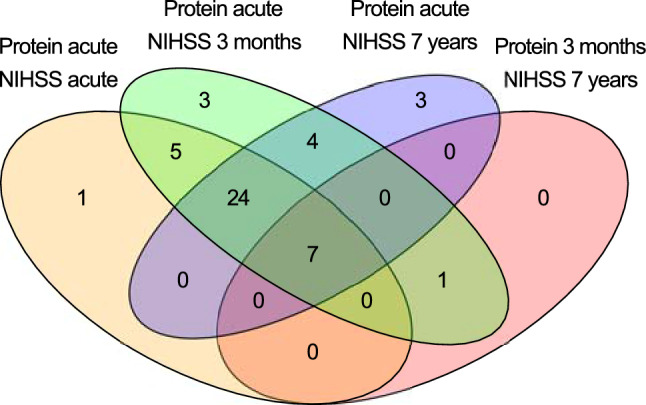
Protein level associations with stroke severity and neurological outcome at the different time points. Number of proteins with a statistically significant association (FDR < 0.05) between plasma levels and degree of neurological impairments according to the NIH stroke scale at the different time points, in linear regression models adjusted for age, sex, and time until blood draw.

Results from the multivariable linear regression analyses for associations between protein levels, acute stroke severity and neurological outcome after adjustment for age, sex and day of blood draw (model 1) are displayed in Figs. 1 and 2, and detailed in Supplemental Table S2. Acute phase levels of 37 proteins were associated with acute stroke severity at FDR < 0.05, and a further 7 proteins were suggestively associated (*p* < 0.05, FDR ≥ 0.05). Acute phase levels of 44 proteins were significantly associated with 3-month neurological outcome, and a further 5 proteins were suggestively associated. For a vast majority of these proteins, lower plasma levels were associated with higher NIHSS scores, i.e. worse stroke severity/outcome, and the acute phase plasma levels of these proteins were also generally lower than in controls (Fig. 1, Panels A, B and E).

For associations between protein levels in the acute phase and long-term outcome (7-year NIHSS) a similar pattern emerged; 38 proteins were associated to 7-year neurological outcome at FDR < 0.05 and a further 11 were suggestively associated. Similar to the analyses of 3-month outcome, most of these associations were inverse and there was a high overlap of proteins associated to both 3-month and 7-year neurological outcome (Figs. 1,2).

When additionally adjusting the models for stroke severity (acute NIHSS; model 2), the signals were attenuated and no association withstood correction for multiple testing at either time point (Supplemental Tables S2). However, levels of 15 proteins were suggestively associated with 3-month outcome and 5 proteins with 7-year neurological outcome.

### Three-month levels of Brevican core protein (BCAN) and Neuronal cell adhesion molecule (Nr-CAM) associated to long-term neurological outcome after adjustment for acute stroke severity and multiple testing

Three-month levels of 8 proteins were associated to 7-year NIHSS after adjustment for age, sex and sampling day (model 1) at FDR < 0.05, and a further 17 proteins were suggestively associated (*p* < 0.05, FDR ≥ 0.05; Fig. 1 and Supplemental Table S2). After additional adjustment for acute stroke severity (acute NIHSS; model 2), 3-month levels of BCAN and Nr-CAM were significantly associated to 7-year neurological outcome (fully adjusted model: β = − 1.38, 95% CI, − 2.13 to − 0.63, *p* = 3.5 × 10^−4^, FDR = 0.032, and β = − 2.49, 95% CI, − 3.96 to − 1.03, *p* = 9.5 × 10^−4^, FDR = 0.043, respectively), and 16 additional proteins showed suggestive associations (*p* < 0.05, FDR ≥ 0.05, Supplemental Table S2). Three-month levels of BCAN and Nr-CAM were correlated at r = 0.54, *p* = 4.0 × 10^−17^, and when entering both in the same model together with age, sex, sampling day and acute stoke severity (acute NIHSS), associations with 7-year neurological outcome were attenuated for both proteins (BCAN: β = − 0.97, 95% CI, − 1.87 to − 0.078, *p* = 0.033; Nr-CAM β = − 1.45, 95% CI, − 3.19 to 0.29, *p* = 0.10).

### Cross-sectional analyses of 7-year protein levels and neurological outcome

At 7 years, levels of 9 proteins were associated to NIHSS scores measured at the same time point with FDR < 0.05 after adjustment for age, sex and sampling day (model 1), and an additional 11 proteins showed suggestive associations (*p* < 0.05, FDR ≥ 0.05). All but one were previously associated with outcome (*p* < 0.05) also at an earlier sampling point (Supplemental Table S2).

### Sensitivity and stratified analyses

When excluding cases with recurrent stroke during follow-up and/or neurological comorbidities (n = 29), similar associations to neurological outcome were generally observed across all time-points (Supplemental Table S3). The associations between protein levels and NIHSS scores in the acute phase and at 3 months, were generally more pronounced in cases with anterior non-lacunar strokes (n = 67 cases with TACI or PACI, Supplemental Table S4).

### Secondary outcome analyses; modified Rankin Scale (mRS)

In the secondary outcome analyses, 32 acute-phase proteins were significantly associated to 3-month functional outcome (mRS 0–2 vs mRS > 2; FDR < 0.05; model 1) and an additional 7 proteins were suggestively associated (Supplemental Table S5). All of these associations were directionally consistent with the 3-month primary outcome (NIHSS) analyses. For the remaining models and time points, there were no proteins significantly associated to functional outcome (mRS; Supplemental Table S5). However, in each model there were suggestive associations to mRS that were concordant to the NIHSS analyses results (*p* < 0.05 and same direction in both). Examples include: acute-phase levels and 3-month outcome, 1 protein (Repulsive guidance molecule A [RGMA]); acute-phase levels and 7-year outcome, 3 proteins (Galectin-8 [gal-8], Dipeptidyl peptidase 1 [CTSC] and Testican-1 [SPOCK1]); 3-month levels and 7-year outcome, 5 proteins (BCAN, Mitochondrial hydroxyacylglutathione hydrolase [HAGH], CTSC, Neurocan core protein |NCAN], and Mesencephalic astrocyte-derived neurotrophic factor [MANF]); and 7-year levels and 7-year outcome, RGMA.

### Temporal patterns

**Figure 3:**
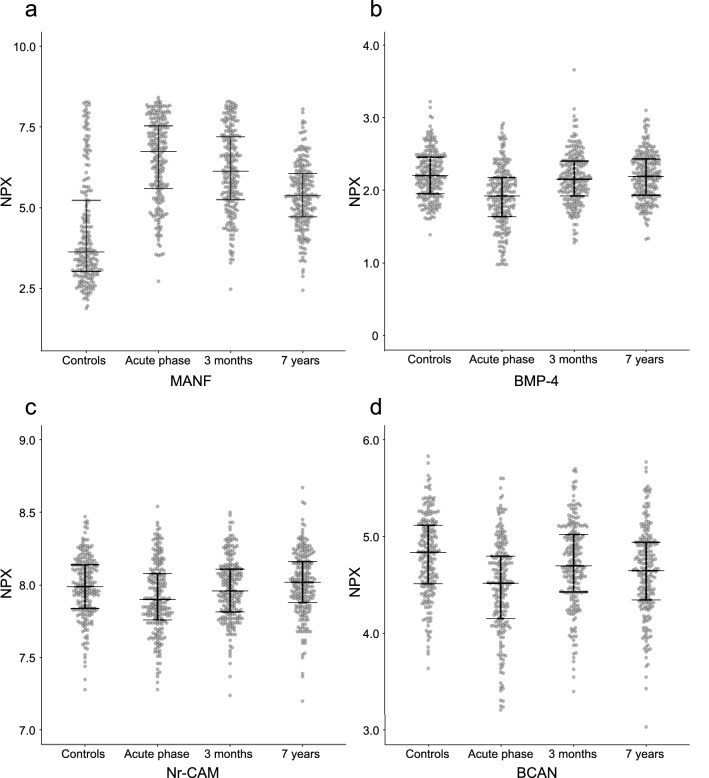
Selected temporal patterns of plasma proteins levels. Distribution as well as median and interquartile range of protein levels according to Normalized Protein eXpression (NPX) levels. Each dot represents one individual sample. (**a**) The first temporal pattern is represented by Mesencephalic astrocyte-derived neurotrophic factor (MANF), where higher plasma levels in ischemic stroke cases than controls are noted in the acute phase followed by a subsequent decrease over time in cases (*p* < 10^−4^, FDR < 10^−4^ for difference throughout). (**b**) The second pattern is represented by Bone morphogenetic protein 4 (BMP-4), where lower levels are observed in the acute stroke phase (*p* = 1.18 × 10^−14^, FDR = 1.54 × 10^−13^ for difference vs. control levels, and *p* = 7.30 × 10^−16^, FDR = 3.16 × 10^−15^ compared to 3-month levels), followed by a subsequent normalization at 3-months and thereafter (*p* > 0.20 throughout). (**c**) Temporal patterns for Neuronal cell adhesion molecule (Nr-CAM) and (**d**) Brevican core protein (BCAN). For both Nr-CAM and BCAN, levels were statistically significantly altered in the acute stroke phase compared to controls, and between follow-up evaluations within cases (*p* < 0.001, FDR < 0.01 throughout).

Among the stroke cases, mean levels of 75 proteins showed statistically significant (FDR < 0.05) differences between the acute and the 3-month sampling points, of which n = 57/75 (76%) were lower in the acute phase. Between the 3-month and 7-year sampling points, mean levels of 50 proteins differed, of which n = 39/50 (78%) were lower at 3-months. Ratios and correlations between protein levels at the different time points are given in Supplemental Table S6. The temporal pattern, and control levels for comparisons, are displayed for 4 selected examples in Fig. 3, Panels A–D and results are detailed in Supplemental Tables S6, S7. Of note, most proteins associated to outcome followed a trajectory with lower levels in the acute stroke phase compared to the convalescent phase and the single sampling point for the controls. For these proteins, higher levels in cases were generally associated with less degree of neurological deficits at follow-up. However, there were differing examples; for instance, levels of MANF and HAGH were higher in the acute and convalescent phase, with *higher* 3-month levels suggestive for association to less favourable 7-year neurological and functional outcome after adjustment for acute stroke severity. In contrast, levels of CTSC, another protein with suggestive association to long-term outcome above acute stroke severity, remained fairly stable during follow-up in cases (*p* > 0.28 for difference throughout) with a trend towards higher levels in cases than controls at 3 months post stroke (*p* = 0.031, FDR = 0.073, Supplemental Table S6, S7).

### Protein clusters and correlations to clinical variables; few strong correlations between proteins levels and cardiovascular risk factors

**Figure 4:**
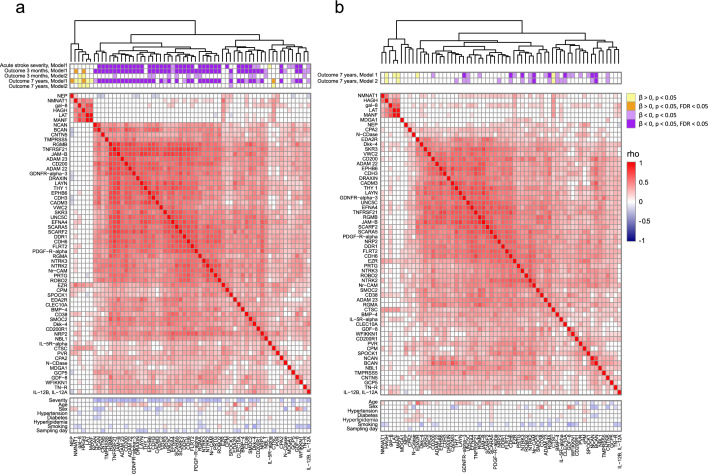
Acute phase and 3-month protein clusters, and correlations with clinical variables for 62 candidate acute and/or 3-month markers with statistically significant or suggestive associations with acute stroke severity or neurological outcome. Hierarchical protein clusters based on Euclidean distance in the acute phase (**a**) and at 3-months after stroke (**b**), with information on association between protein levels (Normalized Protein eXpression [NPX]) and neurological outcome according to the NIH stroke scale (NIHSS) given in the first rows. Model 1 includes adjustment for age, sex and day of blood draw and model 2 is additionally adjusted for acute stroke severity (NIHSS). In following rows, Spearman’s rank correlation coefficients (r) between proteins and clinical variables at each time-point are displayed in heat maps where correlations with *p* < 0.05 are marked in colour. Positive correlations (r > 0) between protein levels, increasing stroke severity (NIHSS), age, and sampling day are marked red, and inverse correlations (r < 0) are marked blue. For sex, a positive correlation (r > 0, red boxes) corresponds to higher protein levels in males than females, and for remaining risk factors a positive correlation corresponds to higher protein levels in participants with that risk factor prevalent.

**Figure 5:**
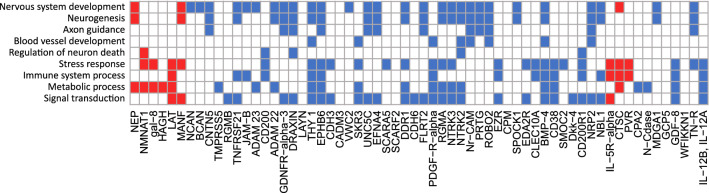
Selected annotated biological functions for 62 candidate acute and/or 3-month protein markers with either statistically significant or suggestive association with acute stroke severity or neurological outcome. Present annotations according to the Gene Ontology (GO) database^18^, version released July 1, 2022, doi: 10.5281/zenodo.6799722. Proteins are sorted by hierarchical clustering according to Euclidean distance in the acute phase. Annotations are marked in blue for proteins where lower plasma levels in the acute stroke phase were associated with higher NIHSS scores (worse stroke severity or outcome), and red for proteins for which higher plasma levels were associated with higher NIHSS scores.

For the 62 proteins for which associations between acute levels and stroke severity or neurological outcome, or 3-month levels and 7-year neurological outcome were either statistically significant or suggestive in at least one model, correlations between protein levels and cardiovascular risk factors are displayed in Fig. 4 and selected biological function annotations are given in Fig. 5. Of note, there was more than one cluster of highly covariating proteins at both time-points, and proteins associated with outcome were represented across these clusters (Fig. 4). In the acute phase, proteins within the largest cluster were generally inversely correlated to acute stroke severity (NIHSS), and less often to sampling day (Fig. 4, panel A, and Supplemental Table S8).

Protein levels were often correlated to age and sex (Fig. 4 and Supplemental Table S8). The variance in protein levels explained by age, sex and acute stroke severity (model R^2^, linear regression) was generally modest (median R^2^: 0.062 [acute phase], 0.032 [3-months], 0.047 [7-years]). However, at each time point there were exceptions (4 proteins [acute phase], 3 proteins [3-months], 7 proteins [7-years] with R^2^ > 0.2; Supplemental Table S9). We observed few strong correlations to cardiovascular risk factors, and this was true for both cases and controls (Fig. 4, and Supplemental Table S9).

## Discussion

We conducted a study of 91 proteins implicated in neurobiological processes, such as regulation of neuronal death, neurogenesis and axon guidance, in a cohort of 209 acute ischemic stroke survivors and identified a large number of candidate plasma biomarkers of both stroke severity and outcome. Our results add novel information to the field, as the majority of these proteins have not been previously investigated in a clinical stroke study. We also observed signs of processes influencing neurological outcome independent of acute stroke severity, as acute or 3-month levels of 30 proteins were nominally associated to outcome after adjustment for acute NIHSS scores, whereof BCAN and Nr-CAM remained significant also after correction for multiple statistical testing. As the associations to neurological outcome for individual proteins varied by time and across the main observed protein clusters, it is likely that our results reflect more than one biological process with different temporal profiles in response to the ischemic event.

The protein panel used here has not been previously explored in ischemic stroke, but used to investigate plasma biomarkers of neurodegenerative disorders, such as Alzheimer’s disease and atypical parkinsonian syndromes^19–22^. A majority of proteins reported to be associated to these traits also associated with stroke severity and/or outcome in our study, indicating a broader role in pathophysiological processes of neurological disease for these proteins. We also observed potential infarct-induced changes in plasma levels of many of the investigated proteins. First, the differences in plasma levels in ischemic stroke cases compared to the age and sex matched controls were more pronounced in the acute stroke phase than at the 3-month follow-up, and we did not observe strong correlations to traditional cardiovascular risk factors. Also, for ~ 50% of the proteins, acute phase levels correlated to clinical stroke severity, i.e. a proxy for infarct size. As no cases received recanalization therapy, the inverse correlations mostly observed were not due to proteolysis induced by intravenously administered recombinant tissue-type plasminogen activator (t-PA). In addition, the associations between protein levels and acute phase NIHSS scores were generally more pronounced in cases with anterior non-lacunar strokes, possibly due to the NIHSS being more reflective of infarct size in this group. Taken together, this points to our results reflecting endogenous, infarct-induced changes, and a potential for these proteins to aid in our growing understanding of pathways influencing stroke outcome in humans.

Interestingly, most proteins associated with outcome followed a trajectory differing from more well-studied stroke outcome blood biomarkers, e.g. copeptin and NfL, as levels were generally *lower* in the acute stroke phase, and as *lower* acute and 3-month levels were associated with less favorable outcome. Whereas associations between lower circulating protein levels and less favorable outcome have been reported for BDNF, results on correlations between acute BDNF levels and stroke severity have been conflicting^9,10^, suggesting a potential difference compared to candidate proteins identified in our study that were generally inversely correlated to acute phase NIHSS scores. Although the regulation of plasma levels of the proteins investigated in this study is generally unknown, especially in a post-stroke setting, early down-regulation of genes and proteins involved in neuron projection and/or synaptic function has been reported in transcriptomic and proteomic analyses of brain tissue after experimental stroke^23–25^. A subsequent window in the sub-acute stroke phase characterized by upregulated neuronal plasticity and angiogenic pathways is known^26–28^. Conceivably, our results might reflect inter-individual differences in such processes, in turn influencing the post-stroke clinical course.

From a pathophysiological perspective, proteins associated with neurological post-stroke outcome are of interest to explore regardless of ability to add prognostic information above clinical acute stroke severity. In our study, there was a high degree of overlap between proteins covariating with acute stroke severity and outcome, and protein associations to outcome were generally attenuated after additional adjustment for acute NIHSS scores as a consequence thereof. Alternatively viewing stroke severity as a very early outcome, proteins associated to this measure could point to neuroprotective mechanisms in the very early post-stroke phase, e.g. pathways influencing resilience to hypoxia, risk of complications such as hemorrhagic transformation, edema, susceptibility to infections, and more speculatively also to very early neurorestorative processes. For instance, interacting OX-2 membrane glycoprotein (CD200) and Cell surface glycoprotein CD200 receptor 1, both associated to acute stroke severity and neurological outcome in the present study are involved in post-stroke inflammation^29,30^. Similarly, interference with BDNF/NT-3 growth factors receptor (NTRK2) signalling can impact infarct size, neurogenesis and outcome after stroke in vivo^31–33^. Additional examples are given in Table 2 that includes candidate acute and 3-month protein markers significantly associated to neurological outcome in the present study, for which experimental ischemic stroke models provide evidence or suggestion of a causal influence on functional outcome. Of note, among the entire list of proteins for which acute or 3-month levels significantly or suggestively associated to stroke severity or outcome in our study (n = 62 proteins in total), many are currently known to be involved in biological processes of interest from a stroke recovery perspective, e.g. neurogenesis, axon guidance, blood vessel development and regulation of neuronal death (Fig. 5). Whether our results could be explained by inherent differences between cases in such pathways, in turn causally influencing stroke outcome is an important question for future studies, that cannot be deduced from the present study. However, in a recent genome wide association study of neurological instability within the first 24 h after ischemic stroke (n = 5,876) a 2q33.3 loci including both *ADAM23* and *NRP2* reached genome-wide significance, with indication of *ADAM23* driving the association^34^. Acute phase levels of ADAM 23 (Disintegrin and metalloproteinase domain-containing protein 23) were associated to both acute stroke severity, 3-month and 7-year neurological outcome in our study, leading us to hypothesize that causal associations of importance in the early post-stroke phase might be represented among our findings.

**Table 2:** Candidate acute and 3-month protein markers statistically significantly associated to neurological outcome in the present study, for which experimental ischemic stroke models provide evidence or suggestion of a causal influence on functional outcome.

Protein	Full protein name	Gene, localisation	Highest expression (GTEx)	Ischemic stroke outcome study				
				Animal model				Clinical study
				Knockout	Induced overexpression or agonistic treatment	Induced inactivation, down-regulation or inhibition	Other	
**Proteins inversely associated to the NIH Stroke Scale**								
NTRK2	BDNF/NT-3 growth factors receptor *Common alias:* Tropomyosin-Related Kinase B (TrkB)	*NTRK2,* 9q21	Brain		Improved neurological recovery^31,32^, reduced infarct size^31^		Interference in isoform processing: improved neurological outcome, reduced infarct size^33^	Candidate *NTRK2* SNPs associated to neurological outcome^35^ and post-stroke depression^36,37^. Low blood levels of BDNF (ligand) associated to poor functional outcome^2,8–10^
							Treatment with Neurotrophin-3 (ligand): improved sensorimotor recovery^38,39^	
NTRK3	NT-3 growth factor receptor	*NTRK3,* 15q25	Arterial tissue					
DDR1	Epithelial discoidin domain-containing receptor 1	*DDR1,* 6p21	Brain			Improved neurological outcome, reduced infarct size^40^	Treatment with Imatinib (non-selective inhibition): improved neurological outcome^41^, varying effects on infarct size^41–43^	Imatinib added to iv thrombolysis: possible effect on functional outcome (phase II trial)^44^. Phase III ongoing (Clinical Trials/NCT03639922)
PDGF-R-alpha	Platelet-derived growth factor receptor alpha	*PDGFRA,* 4q12	Ovary					
RGMA	Repulsive guidance molecule A	*RGMA,* 15q26	Esophagus			Improved neurological outcome^45,46^, reduced infarct size^45^		
BCAN	Brevican core protein	*BCAN,* 1q23	Brain	Four-gene KO: delayed sensorimotor recovery within first 14 days, no effect on infarct size^47^				
NCAN	Neurocan core protein	*NCAN,* 19p13	Brain				Direct delivery (infarct cavity): no effect on functional outcome^48^	
CD38	ADP-ribosyl cyclase/cyclic ADP-ribose hydrolase 1	*CD38,* 4p15	Spleen	Improved neurological recovery, reduced infarct size^49^		Day 5 intraventricular siRNA administration: Poorer neurological outcome, no effect on infarct size^50^		
GDF-8	Growth/differentiation factor 8	*MSTN,* 2q32	Cervix			Improved skeletal muscle restoration and motoric recovery^51^		
CD200R1	Cell surface glycoprotein CD200 receptor 1	*CD200R1,* 3q13	Spleen	Poorer neurological outcome, no effect on infarct size^30^		Inhibited functional recovery^52^		
**Protein positively associated to the NIH stroke scale**								
NMNAT1	Nicotinamide/nicotinic acid mononucleotide adenylyl-transferase 1	*NMNAT1,* 1p36	Thyroid gland		Improved outcome, reduced infarct size^53,54^	Increased infarct size^54^		

*SNP* Single Nucleotide Polymorphism, *KO* Knockout, *iv* intravenous, *siRNA* Small interfering RNA.

Our results indicate Brevican core protein (BCAN) and Neuronal cell adhesion molecule (Nr-CAM) as candidate 3-month plasma markers of long-term neurological outcome independent of acute stroke severity. Plasma levels of BCAN and Nr-CAM were correlated at r≈0.5 throughout follow-up, suggesting that these proteins in part reflect shared biological processes when measured in plasma.

To the best of our knowledge, plasma levels of BCAN and Nr-CAM have not been previously investigated in a stroke cohort, but others have used the same protein panel as us in search of plasma biomarkers of other central nervous system (CNS) phenotypes. It is interesting to note that BCAN has been among the *top panel candidates* for association to healthier white matter, brain volume and cognitive function^21,55,56^, indicating BCAN as a marker with capacity to reflect these traits in blood. In our study, the difference in BCAN levels between cases and controls was most pronounced in the acute phase, but remained at 3 months. In cases, levels were inversely associated with NIHSS at all time-points including the cross-sectional 7-year assessment. Our results thus suggest both a partially reversible, infarct-induced reduction (supported by experimental studies^57,58^) but also continuously lower BCAN levels in cases not reaching complete neurological recovery. Taken together, this indicates a need to explore if lower BCAN levels also associate with post-stroke cognitive impairment and whether BCAN has a causal role in brain resilience and/or stroke recovery. In the CNS, BCAN is found both in the general extracellular matrix (ECM) and in perineuronal nets (PNN)^59,60^. The latter is a specialized ECM structure that can regulate synaptic transmission and neuronal plasticity^60^ and provide neuronal protection against excitotoxic and oxidative stress^61–63^. Knockout of BCAN and three other PNN constitutes (NCAN, tenascin-R [TN-R] and tenascin-C) lead to delayed sensorimotor recovery within the first 14 days after experimental stroke^47^. Of these, NCAN and TN-R were also evaluated in our study; NCAN-levels were highly correlated with BCAN across follow-up (r≈0.7 throughout), and also associated to outcome at more than one time-point. Acute levels of TN-R were suggestively associated to neurological outcome after adjustment for acute stroke severity. Interestingly BCAN, NCAN and TN-R colocalize with Nr-CAM at the nodes of Ranvier^60^. Further meriting these proteins for in-depth study in the context of recovery after stroke, an intronic variant in the *NRCAM* gene was suggestive for association to white matter hyperintensity volume^64^, and an intronic variant near *TNR* to functional outcome^65^ in two separate genome wide association studies of ischemic stroke cases (n = 3,670 and n = 6,165 respectively). Also, in a targeted expression analysis of brain tissue from six deceased patients, *NRCAM* was up-regulated in areas with signs of angiogenesis^66^.

Strengths of the current study are the inclusion of consecutive and well-characterized ischemic stroke cases with repeated, standardized blood samplings and outcome evaluations across a long-term follow-up. Results were highly concordant using two standardized, clinically broadly used measures of outcome. The protein levels from the different time points were measured simultaneously, using a technology with high specificity^16^. We also undertook measures to limit the impact of run-to-run variability and adjusted for multiple statistical testing using FDR. Moreover, repeated measurements of these proteins over 2 years in 90 healthy individuals showed a high within-individual stability in plasma levels over time for the majority of proteins^67,68^. Our study also has important limitations. Foremost, given the explorative nature of this study, the results require future replication in independent cohorts with repeated blood samplings and outcome measures as they become available. We aimed to identify biomarkers of long-term neurological outcome and our study sample accordingly comprised stroke survivors who attended a 7-year follow-up visit. Consequently, the included cases had fairly mild strokes, which impacted the statistical power in the secondary analyses of functional outcome (mRS), limits the generalizability of our results and signifies the need for future investigation in stroke cohorts with different case-mixes. Similar, since the SAHLSIS study was designed to investigate stroke before 70 years of age, our results cannot be extrapolated to stroke occurring at older ages. Another important aspect is that plasma levels cannot be directly translated into levels in the CNS or cerebrospinal fluid and the mechanism by which many of the investigated proteins reaches the circulation needs to be established for a comprehensive understanding. Finally, to account for more in-depth temporal trajectories and potential protein level-treatment interactions, a future study design with closer clinical evaluations and blood samplings within the first months after stroke would be of interest.

In conclusion, we identified several candidate plasma biomarkers of stroke severity and neurological outcome within a protein panel targeted towards neurobiological processes. We see a potential for these proteins to aid in the search of biological explanations to inter-individual variance in stroke outcomes. Notably, most candidates followed a trajectory differing from previously well-studied markers, as they were lower in the acute phase of ischemic stroke, inversely correlated to acute NIHSS scores, and as lower levels were associated with less favorable outcome. Validation in independent stroke cohorts with high clinical representativeness, ideally closer temporal follow-ups, and additional mechanistic characterization in experimental studies are warranted.

## Supplementary Information

Supplementary Information.

## Data Availability

Summary statistics are provided in the Supplemental information. The full data set is not publicly available, but additional data can be obtained upon reasonable request to the authors.
